# MicroRNA expression profiling of porcine mammary epithelial cells after challenge with *Escherichia coli* in vitro

**DOI:** 10.1186/s12864-017-4070-2

**Published:** 2017-08-24

**Authors:** A. Jaeger, F. Hadlich, N. Kemper, A. Lübke-Becker, E. Muráni, K. Wimmers, S. Ponsuksili

**Affiliations:** 1 Institute for Genome Biology, Leibniz Institute for Farm Animal Biology (FBN), Wilhelm-Stahl-Allee 2, D-18196 Dummerstorf, Germany; 2 Institute for Animal Hygiene, Animal Welfare and Farm Animal Behaviour, University of Veterinary Medicine Hannover, Foundation, D-30559 Hannover, Germany; 3 Institute of Microbiology and Epizootics, Department of Veterinary Medicine at the Freie Universität Berlin, D-14163 Berlin, Germany

**Keywords:** Coliform mastitis, Immune response, Mammary epithelial cells, microRNA, Target genes

## Abstract

**Background:**

Coliform mastitis is a symptom of postpartum dysgalactia syndrome (PDS), a multifactorial infectious disease of sows. Our previous study showed gene expression profile change after bacterial challenge of porcine mammary epithelial cells (PMECs). These mRNA expression changes may be regulated through microRNAs (miRNAs) which play critical roles in biological processes. Therefore, miRNA expression profile was investigated in PMECs.

**Results:**

PMECs were isolated from three lactating sows and challenged with heat-inactivated potential mastitis-causing pathogen *Escherichia coli* (*E. coli*) for 3 h and 24 h, in vitro. At 3 h post-challenge with *E. coli*, target gene prediction identified a critical role of miRNAs in regulation of host immune responses and homeostasis of PMECs mediated by affecting pathways including cytokine binding (miR-202, miR-3277, miR-4903); IL-10/PPAR signaling (miR-3277, miR-4317, miR-548); and NF-ĸB/TNFR2 signaling (miR-202, miR-2262, miR-885-3p). Target genes of miRNAs in PMECs at 24 h were significantly enriched in pathways associated with interferon signaling (miR-210, miR-23a, miR-1736) and protein ubiquitination (miR-125, miR-128, miR-1280).

**Conclusions:**

This study provides first large-scale miRNA expression profiles and their predicted target genes in PMECs after contact with a potential mastitis-causing *E. coli* strain. Both, highly conserved miRNAs known from other species as well as novel miRNAs were identified in PMECs, representing candidate predictive biomarkers for PDS. Time-dependent pathogen clearance suggests an important role of PMECs in inflammatory response of the first cellular barrier of the porcine mammary gland.

**Electronic supplementary material:**

The online version of this article (doi:10.1186/s12864-017-4070-2) contains supplementary material, which is available to authorized users.

## Background

Coliform mastitis (CM) represents one of the most important cardinal symptoms of postpartum dysgalactia syndrome (PDS) in sows [1]. This multifactorial infectious disease has high economic relevance for the pig industry, because both postpartum sows and their piglets can be critically affected by mastitis and lactation failure [2]. Husbandry and management conditions, pathogen contamination, and genetic predisposition are suspected etiological factors that contribute to PDS [3]. Gram-negative pathogens such as *Escherichia coli* (*E. coli*) are most commonly isolated from milk of PDS-positive sows, but are found in non-affected sows as well [4, 5]. Therefore, it is unclear which determinants result in development of subclinical or clinical signs of infection within 12–48 h postpartum, or otherwise preserve clinical health.

Lipopolysaccharide (LPS) endotoxin is a pathogenic factor of *E. coli* and can induce acute and severe inflammation in sows and other animal species [6]. Our previous study showed that *E. coli* induce a fast and strong inflammatory response in porcine mammary epithelial cells (PMECs) [7]. In these cells, bacterial challenge strongly upregulates mRNA expression of genes encoding cytokines, chemokines, and cell adhesion molecules. Furthermore, these changes suggest rapid activation of other immune-competent cells mediating pathogen clearance and host homeostasis through interleukin 1 beta (IL1B) and tumor necrosis factor (TNF) expression [7]. Therefore, cellular as well as molecular factors may be highly relevant to animals’ susceptibility to intramammary infection and mastitis.

MiRNAs are small non-coding RNA molecules (∼22 nucleotides long) and are highly conserved across higher eukaryotic species and can modulate host immune responses [8] as well as are involved in a variety of signaling pathways and biological processes, such as development, cell proliferation, cell differentiation, cell death, metabolism, and disease [9].

In epithelial cells, miRNAs can modulate production and release of cytokines/chemokines and expression of adhesion and costimulatory molecules [10]. According to different microbe-associated molecular patterns (MAMPs) from bacteria, innate and adaptive host immune responses are activated through stimulation of pattern recognition receptors (PRRs) [11]. In bovine mammary epithelial cells, *E. coli* affect expression of miR-184, miR-24-3p, miR-148, miR-486, and let-7a-5p, which target genes involved in developmental/cellular processes, biological regulation, cell growth, and death [12]. Furthermore, miR-142-5p, miR-223, miR-2898, miR-16, and miR-181a are potential markers for mastitis in cattle [12–16].

In contrast, the function of miRNAs in innate and adaptive immunity of porcine mammary glands has not yet been described. Therefore, our study focused on miRNA expression changes in PMECs induced by a potential mastitis-causing *E. coli* strain isolated from milk of PDS-positive sows. We characterized miRNA expression differences in PMECs at 3 h and 24 h after challenge with heat-inactivated *E. coli* to investigate and to compare early and late phase of immune response since specific kinetics and extents of global changes in the transcriptome of bovine mammary epithelial cells were found in a study with comparable experimental design [17]. The response of PMECs was determined by comparing miRNA expression profiles between pathogen-challenged and unchallenged control groups. Together with our previous mRNA expression profiles, miRNA expression data were then integrated based on pairwise correlations and computational target predictions to identify potentially affected signaling pathways. Finally, we performed selective analysis of the most and strongest affected biological processes, molecular functions, and Kyoto Encyclopedia of Genes and Genomes (KEGG)/canonical pathways to investigate the role of miRNAs in pathogen clearance of PMECs.

## Results

### Effects of *E. coli* challenge on miRNA expression in PMECs

MiRNA expression profiling was performed at 3 h post-challenge (hpc) and 24 hpc of PMECs with *E. coli* in comparison to unchallenged control cells using Affymetrix GeneChip miRNA 3.0 arrays. After quality control and filtering of raw data, 3277 probesets corresponding to 1332 mature miRNA sequences were further analyzed. Top10 most highly expressed miRNAs (normalized log2 signal intensity >13) in unchallenged PMECs are miR-23a, miR-24, miR-24a, miR-24-3p, miR-27a, miR-31, miR-31b, miR-221, miR-222, and miR-222a. Compared with other published results in the same cell type, some miRNAs which are highly expressed in pig, were also found as highly expressed in cattle and mice, e.g. miR-26a, miR-205, miR-221, and miR-222 [12, 18, 19]. Furthermore, miR-21, miR-22-3p, miR-27b, miR-27a-3p, miR-31, miR-92a, miR-182, and miR-191 are highly expressed in porcine as well as bovine mammary epithelial cells [12, 18].

**Fig. 1:**
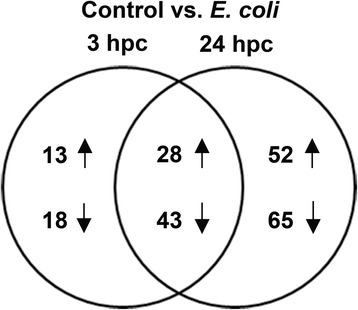
Differentially expressed mature miRNAs following pathogen stimulus. Venn diagram showing miRNAs DE in PMECs at 3 hpc and 24 hpc with *E. coli* (*p* < 0.05; fold change >1.5 or < −1.5; *N* = 3), compared to unchallenged controls. Numbers in the intersecting circles represent miRNAs that were commonly regulated at both challenge times

**Fig. 2:**
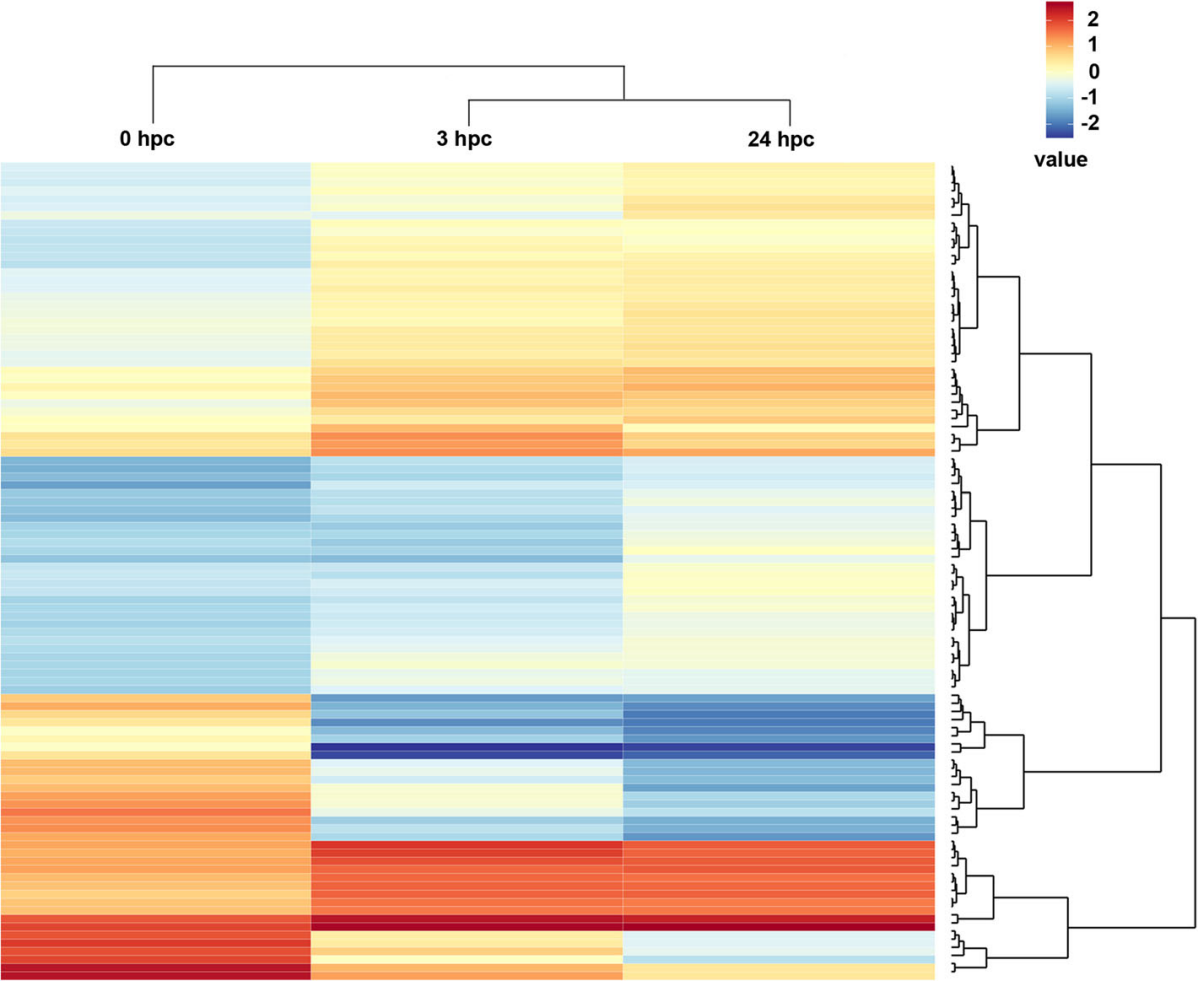
Heat map and hierarchical clustering analysis of the top 100 DE miRNA probesets. Heat map based on processed array signals (Lsmean values log2-transformed; *p* < 0.05; fold change >1.5 or < −1.5) from PMECs at 3 hpc and 24 hpc with *E. coli*, compared to unchallenged controls (0 hpc). Red indicates higher relative expression and blue indicates lower relative expression. Hierarchical clustering dendrogram (ward.D2 method) reveals a distinguishable miRNA expression profile among samples

Statistical analysis identified 143 significantly differentially expressed (DE) probesets (*p* < 0.05; fold change >1.5 or < −1.5) corresponding to 102 mature miRNA sequences (41 up- and 61 downregulated) at 3 hpc with *E. coli* when compared with unchallenged controls (Fig. 1; Additional file 1). At 24 hpc with *E. coli,* 299 significantly DE probesets corresponding to 188 mature miRNA sequences (80 up- and 108 downregulated) were identified as compared to unchallenged controls (Fig. 1; Additional file 1). Approximately 70% of significantly DE miRNAs at 3 hpc with *E. coli* (71 mature miRNA sequences; 28 up- and 43 downregulated) also differed at 24 hpc with *E. coli* (Fig. 1; Additional file 1). In addition, 31 mature miRNA sequences (13 up- and 18 downregulated) were significantly DE only at 3 hpc with *E. coli*. In contrast, 117 mature miRNA sequences (52 up- and 65 downregulated) were significantly DE only at 24 hpc with *E. coli* (Fig. 1; Additional file 1). Some of these miRNAs are not yet annotated in *Sus scrofa*. As concern to this point, we compared these miRNA sequences with *Sus scrofa* miRNA in miRBase (http://www.mirbase.org) and alignment with pig genome (*Sus scrofa* 10.2 download from NCBI: http://www.ncbi.nlm.nih.gov/ on 1.9.2015) and added in Additional file 1 in the column of pig chromosome and position. On the basis of the top 100 significantly DE miRNAs after pathogen challenge, a heat map was generated to identify distinguishable miRNA expression profiles between short-term (3 hpc) and long-term (24 hpc) challenge with *E. coli* compared to unchallenged controls (0 hpc) (Fig. 2). Hierarchical clustering analysis determined similar patterns in miRNA expression profiles of both pathogen-challenged groups compared to unchallenged control group (Fig. 2).

### Prediction of miRNA targets identifies biological processes and molecular functions that differ at 3 hpc and 24 hpc with *E. coli*

Pairwise correlation coefficient analysis was performed to evaluate associations of expression levels between 442 significantly DE miRNA probesets and 1345 significantly DE mRNA probesets (previously identified by Jaeger et al. [7]). In total, 36,610 miRNA–mRNA pairs were significantly negatively correlated (*p* < 0.05) and in silico predicted as functionally linked. TargetScan and RNAhybrid software were used to further filter predicted miRNA–mRNA pairs by setting the threshold for microRNA:target duplex energy to −25 kcal/mol. Finally, we identified 135 miRNA–mRNA pairs corresponding to 34 mature miRNA sequences and 71 genes at 3 hpc with *E. coli* (Table 1; Additional file 2). At 24 hpc with *E. coli*, 531 miRNA–mRNA pairs corresponding to 67 mature miRNA sequences and 214 genes were filtered (Table 2; Additional file 3).

**Table 1:** Top 30 significantly DE miRNAs and their predicted target genes from microarray analysis in PMECs at 3 hpc with *E. coli* compared with unchallenged controls

3 hpc with *E. coli*		
Upregulated miRNA	Sequence (5′ - > 3′)	Downregulated target genes
miR-29b	UAGCACCAUUUGAAAUCAGUGUU	*BCOR, KLHDC7A*
miR-140	CAGUGGUUUUACCCUAUGGUAG	*RAB40B*
miR-210	UUGUGCGUGCGACAGCGACUUC	*RAB40B*
miR-101	UACAGUACUGUGAUAACUGAAG	*EML5, HELLS, RBM12*
miR-29a	UAGCACCAUUUGAAAUCGGUU	*CDC6, CYP26A1, DNAJA1, EML5, RNF2, TIMM44*
miR-24-star	GUGCCUACUGAGCUGAUAUCAGU	*USP46*
miR-30b-star	CUGGGAGGUGGAUGUUUACUUC	*BCOR*
miR-B6	AAGUGCCCGACGCGGGGAACGUG	*ENC1*
let-7 g-star	CUGUACAGGCCACUGCCUUGC	*DUSP6*
miR-125a-3p	ACAGGUGAGGUUCUUGGGAGCC	*ARID5B, ID4, ITGB8, KIAA0182, NUAK1, PHF17, RAB40B, TOX, USP2, VGLL3, WDR35*
Downregulated miRNA	Sequence (5′ - > 3′)	Upregulated target genes
miR-371	ACUCAAAAAAUGGCGGCACUUU	*SGSM1*
miR-548u	CAAAGACUGCAAUUACUUUUGCG	*NFKBIA*
miR-5128	CAAUUGGGGCUGGCGAGAUGGCU	*SGSM1*
miR-8-5p	CAUCUUACCGGGCAGCAUUAGA	*ARHGAP28, HIVEP2, IFNAR2*
miR-259-star	CCACCGAUUUGGCAUGGGAUUGAC	*SGSM1*
miR-3277	UGGGCAUGUUUCUGAAAUUCGAU	*ARHGAP28, IFNAR2, IL1A, PPARG*
miR-4903	UACCCCGGUAGGCAUAGGUUG	*GLRX, IL1RAP, MAP3K8, PLAUR*
miR-202	AGAGGCAUAGGGCAUGGGAAAA	*ARHGAP28, CHL1, GLI3, HIVEP2, IFNAR2, JAG1, LIFR, MAP3K8, OSBPL3, P2RX4, RFFL, RMND5A, RNF19B, TEP1, TNFAIP3, TPM2, VPS37C, YPEL2*
miR-5100	UCGAAUCCCAGCGGUGCCUCU	*OBFC1*
miR-2287	CUGGGACUGGUGGCAGCACUU	*CLDN1, ITGAV, RNF19B*
miR3949	UGAUGUUGAGGCAAAAAUGUAG	*CCDC152, RBM39, RFX1*
miR-885-3p	AGGCAGCGGGGUGUAGUGGAUA	*BIRC3, DCUN1D3, HIVEP2, IL10RB, MAP3K8, TNFAIP3*
miR-2518	UCGAGCAGCGGGUCGAUCCGAGC	*CYP1A1, JAG1*
miR-3042	GAGGGCAGAUUAUUUCUGAUAC	*JAG1, LPIN1, YPEL2*
miR-4031-5p	CCCAAAGUGUCGGCGCAUAU	*SGSM1, TNFAIP3*
miR-4317	ACAUUGCCAGGGAGUUU	*NFKBIA*
miR-216b	AAAUCUCUGCAGGCAAAUGUGA	*ETV5, PDLIM5*
miR-890	UACUUGGAAAGGCAUCAGUUG	*EHF, FCHSD2, IL10RB, PLXND1, SGSM1, SLC4A7, TAP1*
miR394b-star	AGGUGGACAUAUUGCCAACA	*ITGAV, JAG1*
miR-648	AAGUGUGCAGGGCACUGAU	*HIVEP2, IFNAR2, JAG1, SGSM1*

MiRNAs with *p* < 0.05 and fold change >1.5 or < −1.5 are listed and sorted by fold change in descending order. TargetScan-predicted miRNA-mRNA pairs showing negative correlation in expression profile and energy-value of at least −25 kcal/mol

**Table 2:** Top 30 significantly DE miRNAs and their predicted target genes from microarray analysis in PMECs at 24 hpc with *E. coli* compared with unchallenged controls

24 hpc with *E. coli*		
Upregulated miRNA	Sequence (5′ - > 3′)	Downregulated target genes
miR-505	CGUCAACACUUGCUGGUUUCCU	*ABCA7, DDX18, DDX52, EIF4E, GRPEL1, LARP4, MSL1, PLEKHH1, SDC1, SERTAD1, SOCS4, TATDN2, WDR3, YTHDF2, ZBED4*
miR-29a	UAGCACCAUUUGAAAUCGGUU	*CDC6, CYP26A1, DNAJA1, EML5, RNF2, TIMM44*
miR-101	UACAGUACUGUGAUAACUGAAG	*CHD7, EML5, HELLS, LARS, MID1, RBM12*
miR-24-star	GUGCCUACUGAGCUGAUAUCAGU	*USP46*
miR-289	UAAAUAUUUAAGUGGAGCCUGCGACU	*KIAA1430, RNF2, TMCC3*
miR-125-star	ACGGGUUAGGUUCUUGGGAGC	*GIT2, LARS, MSL1, PAFAH1B1, RBM12, SDC1, TMEM79, UBE2R2, ULK3, USP46*
miR-484	UCAGGCUCAGUCCCCUCCCGAU	*FGFR3*
miR-22-star	AGUUCUUCAGUGGCAAGCUUUA	*ARGLU1*
miR-660	UACCCAUUGCAUAUCGGAGCUG	*ARMC8, BCLAF1, CAMKK2, CPT1A, DDX42, MSL1, NOL6, NRARP, PLEKHH1, PPP1R8, PPP6C, PSMD12, PSMF1, PYGO2, RRP12, SDC1, TRIM27, ZFX, ZNF280D*
miR-B6	AAGUGCCCGACGCGGGGAACGUG	*ENC1*
miR-363	AAUUGCACGGUAUCCAUCUGUA	*ABCA7, ADPRH, ATXN1, CAMKK2, DGCR8, FAM53C, GABPB1, GATAD2A, HERPUD2, PAFAH1B1, PLEKHH1, POP4, PPRC1, RBM15, S1PR2, SYK, TATDN2, TFAP2C, TRIM35, TXLNA*
miR-128	UCACAGUGAACCGGUCUCUUUU	*ADPRH, AQP3, GABPB1, KCNJ14, NDNL2, PLEKHH1, RRP12, SDC1, SNX6, TCEB3, UBE2R2, ULK3, ZFAND2A, ZFX, ZNF764*
miR-10a	UACCCUGUAGAUCCGAAUUUGUG	*ADPRHL2, EML5, FARSA, FEN1, IQCC, KLHL18, LMNB2, MFSD9, SFN, SLC16A3, SLC39A6, TATDN2, TLE1, TMCC3, TUBB2B, TXLNA, UBAP2L, ZNF367*
miR-92b	AAUUGCACUAGUCCCGGCCUGC	*CPT1A, GPRC5A, HEXIM1, LIN37, PHF23, PKP1, ULK3, YWHAG*
miR-92b	UAUUGCACUCGUCCCGGCCUCC	*EML5*
Downregulated miRNA	Sequence (5′ - > 3′)	Upregulated target genes
miR-371	ACUCAAAAAAUGGCGGCACUUU	*SGSM1*
miR-4423-3p	AUAGGCACCAAAAAGCAACAA	*RASAL2*
miR5019	UGUUGGGAAAGAAAAACUCUU	*RASAL2*
miR-3128	UCUGGCAAGUAAAAAACUCUCAU	*SLC2A12*
miR5021	UGAGAAGAAGAAGAAGAAAA	*CIRBP*
miR-M9-3p	AAACUCCGAGGGCAGGAAAAAG	*ARHGAP12, ARHGAP28, BIRC3, CEP68, FCHSD2, GRK5, ISG15, LPIN1, MDM4, PCDH7, PDLIM5, SLC2A12, TCTN2, YPEL2*
miR3949	UGAUGUUGAGGCAAAAAUGUAG	*CCDC152, RBM39, RFX1,*
miR-B8-5p	CGCGGGCAAAAAAUCCAAUGGC	*BIRC3, MAP3K8, PLXND1, RNF19B*
miR-2326	CCCCCCUUCCUCUGGAAAAA	*ARHGAP28, CEP68, CHI3L1, CXCL2, FNBP1, LPIN1, PCDH7, PLXND1, PPAP2B, RFFL, RHOQ, SCUBE3, SLC4A7, TPM2*
miR-5100	UCGAAUCCCAGCGGUGCCUCU	*OBFC1*
miR-259-star	CCACCGAUUUGGCAUGGGAUUGAC	*SGSM1*
miR-2779	AUAUCCGGCUCGAAGGACCA	*CLDN1, PLXND1*
miR-4592	CCAGCGGCGGUGCCGUGAUGGCGA	*CLDN1, CEP68, CXCL2, ITGB8, MDM4, RHOQ*
miR-202	AGAGGCAUAGAGCAUGGGAAAA	*ARHGAP28, BIRC3, CHL1, GLI3, LIFR, MAP3K8, P2RX4, PLN, RASAL2, RFFL, RMND5A, RNF19B, SGSM1, TEP1, TNFAIP3, TPM2, VPS37C*
miR-3277	UGGGCAUGUUUCUGAAAUUCGAU	*ARHGAP28, IFNAR2, IL1A, PPARG*

MiRNAs with *p* < 0.05 and fold change >1.5 or < −1.5 are listed and sorted by fold change in descending order. TargetScan-predicted miRNA-mRNA pairs showing negative correlation in expression profile and energy-value of at least −25 kcal/mol

**Fig. 3:**
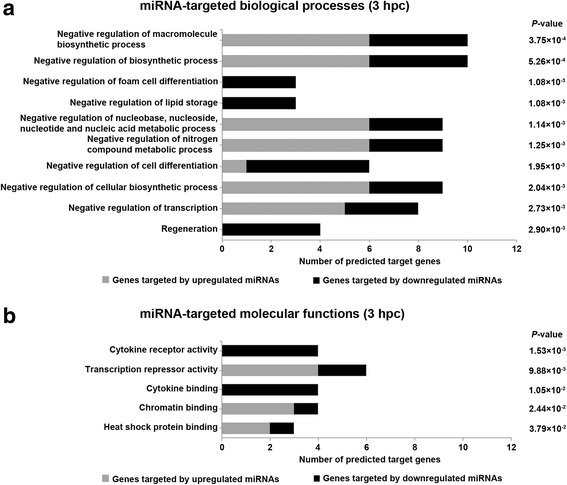
Top categories of biological processes and molecular functions regulated by predicted target genes of significantly DE miRNAs at 3 hpc with *E. coli*. **a** Predicted target genes of upregulated (grey bars) and downregulated (black bars) miRNAs from PMECs at 3 hpc with *E. coli* were significantly enriched in biological processes such as negative regulation of biosynthetic and metabolic processes, transcription, lipid storage, cell differentiation, and regeneration. **b** Predicted target genes of up- and downregulated miRNAs from PMECs at 3 hpc with *E. coli* regulate molecular functions such as cytokine receptor and transcription repressor activity, as well as binding of cytokines, chromatin, and heat shock proteins

Gene ontology (GO) analysis identified biological processes and molecular functions predicted for target genes of DE miRNAs. Predicted target genes of up- and downregulated miRNAs in PMECs at 3 hpc with *E. coli* were significantly associated with negative regulation of biosynthetic processes, metabolic processes, and transcription (*ARID5B*, *BCOR*, *BMP2*, *CDC6*, *GLI3*, *HELLS*, *ID4*, *ITGAV*, *PPARG*, and *RNF2*); lipid storage (*ITGAV*, *NFKBIA*, and *PPARG*); cell differentiation (*GLI3*, *ID4*, *ITGAV*, *JAG1*, *NFKBIA*, and *PPARG*); and regeneration (*JAK2*, *LIFR*, *PLAUR*, and *PPARG*) (Fig. 3a; Additional file 4). Most genes were predicted targets of predominantly miR-101, miR-125a-3p, miR-202, miR-2262, miR-29a, miR-29b, miR-30b-star, and miR-3277 (Additional file 4).

Molecular functions influenced by predicted target genes of up- and downregulated miRNAs in PMECs at 3 hpc with *E. coli* included cytokine receptor activity and cytokine binding (*IFNAR2*, *IL10RB*, *IL1RAP*, and *LIFR*); transcription repressor activity (*ARID5B*, *BCOR*, *BMP2*, *ID4*, *PPARG*, and *RNF2*); chromatin binding (*CDC6*, *GLI3*, *HELLS*, and *RNF2*); and heat shock protein binding (*BCOR*, *DNAJA1*, and *NFKBIA*) (Fig. 3b; Additional file 4). Most genes were predicted targets of predominantly miR-29a, miR-202, and miR-3277 (Additional file 4).

**Fig. 4:**
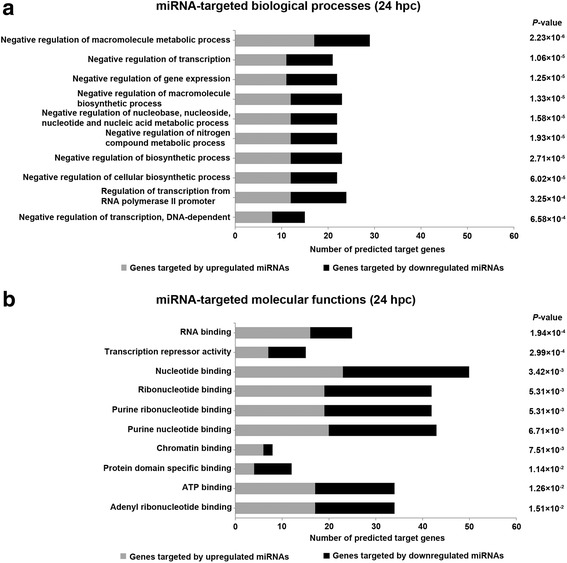
Top categories of biological processes and molecular functions regulated by predicted target genes of significantly DE miRNAs at 24 hpc with *E. coli*. **a** Predicted target genes of upregulated (grey bars) and downregulated (black bars) miRNAs from PMECs at 24 hpc with *E. coli* were significantly enriched in biological processes such as negative regulation of metabolic processes, transcription, gene expression, biosynthetic processes, and regulation of transcription from RNA polymerase II promoter. **b** Predicted target genes of up- and downregulated miRNAs from PMECs at 24 hpc with *E. coli* were significantly enriched in molecular functions such as transcription repressor activity, as well as binding of RNA, nucleotides, ribonucleotides, purine ribonucleotides, purine nucleotides, chromatin, protein domains, ATP, and adenyl ribonucleotides

miRNAs that were up- and downregulated in PMECs at 24 hpc with *E. coli* were negatively correlated with target genes that were also involved in negative regulation of metabolic processes, transcription, gene expression, biosynthetic processes, and regulation of transcription from RNA polymerase II promoter (e. g., *ATF7IP*, *BCOR*, *BMP2*, *HEXIM1*, *ID3*, *MDM4*, *PPARG*, *RBM15*, *RNF2*, *SP100*, *TLE1*, and *TRIM27*) (Fig. 4a; Additional file 4). Most genes were predicted targets of predominantly miR-10a, miR-1934-star, miR-23a-star, miR-263a-star, miR-289, miR-29a, miR-30b-star, miR-3216, miR-3277, miR-34c, miR-363, miR-4592, miR-660, miR-92b, miR-M9-3p, miR-rL1–34-5p, and miR894 (Additional file 4).

Molecular functions influenced by predicted target genes of up- and downregulated miRNAs in PMECs at 24 hpc with *E. coli* included transcription repressor activity (e. g., *ARID5B*, *ATF7IP*, *ATXN1*, *BCLAF1*, *BMP2*, *CREBZF*, *HEXIM1*, *PPARG*, *SP100*, and *TLE1*); binding of nucleotides, ribonucleotides, purine ribonucleotides, purine nucleotides, adenyl ribonucleotides, and ATP (e. g., *ABCA7*, *AKAP9*, *CAMKK2*, *CHD7*, *CLK1*, *FGFR2*, *FGFR3*, *IGF1R*, *TOP1*, and *UBE2R2*); RNA binding (e. g., *ATXN1*, *DDX18*, *DGCR8*, *EIF4E*, *LARP4*, *NOL6*, *OBFC2A*, *RBM41*, *RBM5*, and *RPUSD4*); chromatin binding (*CDC6*, *CHD7*, *GLI3*, *HELLS*, *RNF2*, *SF3B1*, *TLE1*, and *TOP1*); and protein domain specific binding (*BMP2*, *CLDN1*, *ELMO1*, *FUT8*, *ID3*, *LUC7L*, *RHOQ*, *RIPK1*, *SFN*, *SP100*, *SYK*, and *YWHAG*) (Fig. 4b; Additional file 4). Most genes were predicted targets of predominantly miR-10a, miR-1280, miR-1934-star, miR-23a-star, miR-263a-star, miR-363, miR-660, miR-92b, and miR894 (Additional file 4).

The number of target genes predicted for DE miRNAs and involved in the regulation of biological processes or molecular functions in PMECs is about two- to tenfold higher at 24 hpc than at 3 hpc with *E. coli*, respectively.

### Enriched KEGG pathways and canonical pathways of predicted gene targets of DE miRNAs differ at 3 hpc and 24 hpc

**Fig. 5:**
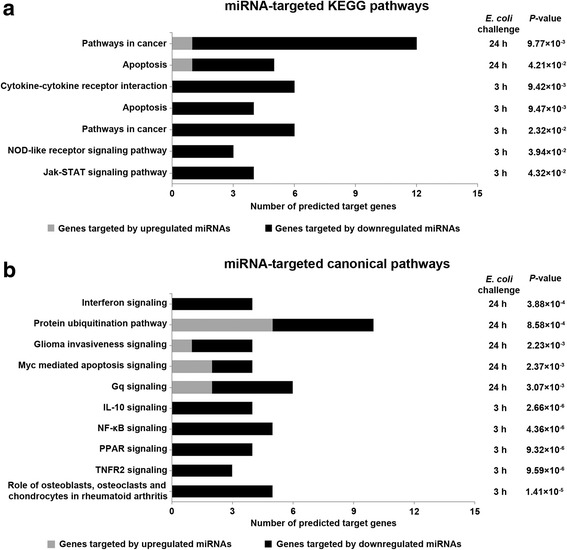
Top KEGG pathways and canonical pathways regulated by predicted target genes of significantly DE miRNAs at 3 hpc and 24 hpc with *E. coli*. **a** Predicted target genes of predominantly downregulated (black bars), but also upregulated (grey bars), miRNAs from PMECs at 3 hpc and 24 hpc with *E. coli* were significantly enriched in KEGG pathways such as cytokine–cytokine receptor interactions, apoptosis, cancer pathways, NOD-like receptor signaling pathways, and Jak-STAT signaling pathways. **b** Predicted target genes of downregulated miRNAs were significantly enriched in canonical pathways such as IL10 signaling, NF-kB signaling, PPAR signaling, TNFR2 signaling, and the role of osteoblasts, osteoclasts, and chondrocytes in rheumatoid arthritis in PMECs at 3 hpc with *E. coli*. Predicted target genes of up- and downregulated miRNAs were significantly enriched in canonical pathways such as interferon signaling, protein ubiquitination, glioma invasiveness signaling, myc-mediated apoptosis signaling, and Gq signaling in PMECs at 24 hpc with *E. coli*

In KEGG analysis, target genes of mostly downregulated miRNAs in PMECs at 3 hpc and 24 hpc with *E. coli* were significantly enriched in pathways in cancer (e. g., *BIRC3*, *BMP2*, *GLI3*, *ITGAV*, *NFKBIA*, and *PPARG*) and apoptosis (e. g., *BIRC3*, *IL1A*, and *NFKBIA*) (Fig. 5a). miR-3277, miR-548u, and miR-885-3p were highly predicted to play important roles in regulating both KEGG pathways (Additional file 5).

At 3 hpc with *E. coli,* target genes of downregulated miRNAs were significantly enriched in cytokine–cytokine receptor interactions (*BMP2*, *IFNAR2*, *IL10RB*, *IL1A*, *IL1RAP*, and *LIFR*); nucleotide-binding oligomerization domain (NOD)-like receptor signaling pathways (*BIRC3*, *NFKBIA*, and *TNFAIP3*); and Janus kinase/signal transducers and activators of transcription (Jak-STAT) signaling pathways (*IL10RB*, *IFNAR2*, *JAK2*, and *LIFR*) (Fig. 5a). miR-202, miR-2262, and miR-885-3p were highly predicted to play important roles in regulating expression of genes that are involved in the three named KEGG pathways. Furthermore, most genes regulating cytokine signaling and Jak-STAT signaling were highly predicted to be targets of miR-3277, miR-648, miR-8-5p, and miR-890 (Additional file 5). Target genes of predominantly downregulated miRNAs in PMECs at 3 hpc with *E. coli* were significantly enriched in canonical pathways such as IL-10 signaling (*IL1A*, *IL10RB*, *IL1RAP*, and *NFKBIA*); NF-ĸB signaling (*BMP2*, *IL1A*, *MAP3K8*, *NFKBIA*, and *TNFAIP3*); peroxisome proliferator-activated receptor (PPAR) signaling (*IL1A*, *IL1RAP*, *NFKBIA*, and *PPARG*); tumor necrosis factor receptor 2 (TNFR2) signaling (*BIRC3*, *NFKBIA*, and *TNFAIP3*); and the role of osteoblasts, osteoclasts and chondrocytes in rheumatoid arthritis (*BIRC3*, *BMP2*, *IL1A*, *IL1RAP*, and *NFKBIA*) (Fig. 5b). miR-3277, miR-4317, miR-4903, miR-548u, and miR-885-3p were highly predicted to be important for regulation of most of the named canonical pathways (Additional file 5).

Target genes of predominantly downregulated miRNAs in PMECs at 24 hpc with *E. coli* were significantly enriched in canonical pathways such as interferon signaling (*MX1*, *ISG15*, *STAT1*, and *TAP1*); protein ubiquitination (*PSMB9*, *BIRC3*, *DNAJA1*, *DNAJC11*, *PSMD12*, *TAP1*, *UBE2R2*, *USP28*, and *USP46*); glioma invasiveness signaling (*DIRAS3*, *FNBP1*, *ITGAV*, and RHOQ); myc-mediated apoptosis signaling (*FAS*, *IGF1R*, *SFN*, and *YWHAG*); and Gq signaling (*DIRAS3*, *FNBP1*, *GNA14*, *HRH1*, *NFKBIA*, and *RHOQ*) (Fig. 5b). miR-210, miR-23a-star, and miR-324-5p were highly predicted to play important roles in regulating most of the named canonical pathways (Additional file 5).

### Validation of selected miRNA expression by real time quantitative PCR

Seven miRNAs (miR-210, miR-423-5p, miR-17-3p, miR-193a-5p, miR-320, miR-339-5p, and miR-362) that were DE in miRNA microarray analyses were selected for validation by real time quantitative PCR (RT-qPCR). MiRNA expression analyzed by RT-qPCR was in good accordance with microarray data, as shown by similar fold changes (FC) (Additional file 6). This suggested that our microarray data were reliable, although we used pools of samples for microarrays compared to individual samples for qPCR.

## Discussion

The role of miRNAs in innate and adaptive immunity of the mammary gland is increasingly well described in different species, e. g. human, mice, and cattle. In pigs, the miRNA role regulating inflammatory defense mechanisms during microbial infection of the mammary gland has not yet been described. Therefore, we used the previously described PMEC model [7] to analyze specific pathogen defense mechanisms under standardized experimental conditions in vitro*.* To the best of our knowledge, this is the first study describing specific miRNAs and their predicted target genes in PMECs after contact with a potential mastitis-causing *E. coli* strain, indicating the important roles of these cells in pathogen clearance within the first cellular barriers of the porcine mammary gland.

DE miRNAs that were identified in PMECs at 3 hpc and 24 hpc with *E. coli* were analyzed together with our previous mRNA expression profile and then integrated based on pairwise correlations and computational target predictions.

Pathogen challenge interfered with many host cellular processes, including modulating miRNA production, which influences host cell physiology and defense. As shown in this study, many miRNAs were significantly upregulated in PMECs at 3 hpc and 24 hpc with *E. coli*. Expression data showed that long incubation of PMECs with heat-inactivated *E. coli* resulted in more DE miRNAs than short incubation. Early response of PMECs at 3 hpc with *E. coli* was followed by a late, more intensive host response at 24 hpc, as indicated by an approximately two-fold increase of significantly DE miRNAs. This is in accordance with our mRNA microarray analysis, which also identified a more intense host response after long-term challenge with *E. coli* [7].

Approximately 46% and 17% of identified mRNAs are predicted to be miRNA-regulated in PMECs at 3 hpc and 24 hpc with *E. coli*, respectively. Some of the identified DE miRNAs have not yet been described in the current literature. But based on our target prediction analysis and existing literature, most of the identified DE miRNAs likely play important roles in regulation of innate immunity and cellular homeostasis of PMECs. Among these, miR-101, miR-24-star, miR-30b-star, miR-210, miR-34c, and miR-17-3p were significantly DE in PMECs after *E. coli* challenge and are also affected during host responses of human, mice, or cattle to LPS stimulus or *E. coli* infection [12, 20–24]. In addition, some DE miRNAs in our analysis play pivotal roles in regulation of host defenses against other bacterial infections (miR-23a-star, miR-125-star, and miR-128) [12, 25, 26]; viral infections (miR-29a, miR-505, and miR-423-5p) [27–29]; parasitization (miR-8-star) [30]; and inflammatory bowel disease (miR-10a and miR-5128) [31, 32]. Furthermore, miR-101, miR-29b, miR-10a, miR-17-3p, and miR-92b were DE expressed in PMECs after pathogen challenge and are important regulators in bovine (endo-) metritis [33]. In addition, miR-484, miR-660, miR-34c, miR-339, miR-193a-5p, and miR-362 have regulatory functions in breast cancer progression [34–39] and were also deregulated in PMECs after pathogen challenge. Likewise, some miRNAs that were DE in PMECs at 3 hpc and 24 hpc with *E. coli* are potential regulators of genes that were enriched in KEGG pathways, such as cancer-related or apoptosis pathways. Therefore, a deregulation of growth regulation or an alteration of cellular physiological processes such as cell proliferation, cell death and repair of genes in PMECs by *E. coli* infection is assumed, which might play a pivotal role in the genesis of PDS.

Staedel and Darfeuille [25] reported that recognition of intruding pathogens by PAMPs — such as membrane-associated TLRs and cytoplasmic NOD-like receptors — is essential for subsequent activation of innate and adaptive immunity, mostly stimulated by NF-ĸB signaling. Accordingly, we identified miR-202, miR-2262, miR-4031-5p, miR-4317, miR-548u, and miR-885-3p as potential regulators of target genes that are enriched in NOD-like receptor signaling pathways in PMECs, referring to recognition of *E. coli*. In contrast, the TLR signaling pathway was not identified as one of the top enriched canonical pathways in PMECs after pathogen challenge as discuss in our previously study [7]. It is possible that the preparation of heat kill particles somehow removed TLR agonists from the particles from pMECs but not from bMECs [40]. This is still lack of knowledge in this field of experiment. However, miR-101, miR-125-star, miR-8-star, and miR-29a were DE in PMECs after pathogen challenge and are involved in TLR signaling [20, 25, 30].

MiRNAs can interact with several signaling molecules, such as cytokines, chemokines, and transcription factors, and therefore influence various signaling pathways and cellular processes [25]. In our study, miR-202, miR-3277, miR-4903, miR-648, miR-8-5p, miR-885-3p, and miR-890 were identified to play critical roles in regulation of genes that control cytokine receptor activity and cytokine binding. Additionally, some miRNAs that were DE in PMECs after pathogen challenge are known to regulate genes that are involved in NF-ĸB signaling (miR-210 and miR-23a-star) [41, 42] or production of cytokines IL-6, TNF-alpha, IL-1 beta, and IL-10 (miR-30b-star, miR-210, miR-17-3p, miR-23a-star, miR-24-star, miR-29b, miR-125-star, miR-1934-star, and miR-320) [21, 22, 24, 42–47]. Furthermore, our analysis identified predicted target genes of DE miRNAs in PMECs at 3 hpc with *E. coli* that are enriched in top canonical pathways such as IL-10 signaling, PPAR signaling, NF-ĸB signaling, TNFR2 signaling, or arthritis-associated roles. These signaling pathways are important for fast pathogen recognition and effective nonspecific removal of infectious agent as well as moderate the inflammatory response to limit tissue damage of the host. At 24 hpc of PMECs with *E. coli*, predicted target genes of DE miRNAs were enriched in top canonical pathways such as interferon signaling and protein ubiquitination, which were also significantly enriched in our previous mRNA analysis of pathogen-challenged PMECs [7]. In this late immune response phase these signaling pathways can increase host-defense by activation of immune cells and regulation of a variety of fundamental cellular processes.

Altogether, the number of target genes predicted for DE miRNAs and involved in regulation of biological processes or molecular functions in PMECs was two- to tenfold higher at 24 hpc than at 3 hpc with *E. coli*.

In addition to our target prediction analysis, current literature suggests that some DE miRNAs in PMECs also regulate genes involved in Jak-STAT signaling (miR-125-star, miR-289, miR-22-star, and miR-23a-star) [45, 48–50]; interferon signaling (miR-29a) [28]; MAPK signaling (miR-289, miR-324-5p, and miR-193a-5p) [48, 51, 52]; TGF signaling (miR-125-star, miR-324-5p, miR-193a-5p, and miR-505) [45, 51–53]; PTEN/Akt signaling (miR-92b) [54]; and Wnt signaling (miR324-5p and miR-193a-5p) [51, 52]. Interaction of signaling molecules that are involved in these pathways seems to be important to balance pro- and anti-inflammatory processes and to ensure immune homeostasis as well as host protection [55]. This is confirmed by our results, as some miRNAs that were DE in PMECs after pathogen challenge play crucial roles in innate and adaptive immune responses (miR-101, miR-125-star, miR-423-5p, miR-10a, miR-23a-star, miR-1934-star, miR-29a, miR-29b, miR-24-star, let-7 g-star, miR-320, miR-17-3p, miR-324-5p, and miR-8-star) [20, 25, 27, 31, 42, 46, 56–64] as well as negative feedback regulation of inflammation (miR-210 and miR-17-3p) [22, 62].

Our target prediction analysis revealed several DE miRNAs as potential regulators of genes that control biosynthetic, metabolic, and transcriptional processes in PMECs at 3 hpc as well as 24 hpc with *E. coli*. In addition, some DE miRNAs in our study are known to target genes involved in regulation of cell adhesion/cytoskeleton (miR-17-3p, miR-339, and miR-8-star) [62, 65, 66]; cell survival/DNA repair (miR-324-5p, miR-8-star, miR-890, miR-885-3p, and miR-363) [63, 65, 67–70]; catabolism (miR-320 and miR-140) [44, 47, 70]; migration/invasion/tumorigenesis (miR-339, miR-193a-5p, miR-362, miR-125-star, miR-92b, miR-324-5p, miR-320, miR-202, miR-371, miR-125a-3p, and miR-140) [37–39, 45, 54, 63, 71–75]; cellular transport (miR-10a and miR-5100 [60, 76]; proliferation/differentiation/cell cycle/growth/development (miR-484, miR-34c, miR-339, miR-193a-5p, miR-362, miR-125-star, miR-92b, miR-24-star, miR-10a, miR-324-5p, miR-363, miR-320, miR-202, miR-371, miR-125a-3p, miR-140, miR-5100, miR-128, miR-216b, miR-4423-3p, and miR-320b) [34, 36–39, 45, 54, 58, 60, 63, 67, 71–80]; and apoptosis/ autophagy/ phagocytosis (miR-30b-star, miR-125-star, miR-324-5p, miR-505, miR-92b, miR-24-star, miR885-3p, miR-320, miR-128, miR-216b, miR-320b, miR-125a-3p, miR-363, and miR-423-5p) [43, 45, 51, 53, 54, 58, 69, 71, 77, 78, 80–84]. These results are consistent with our mRNA study, in which pathogen challenge of PMECs resulted in DE genes that regulate cellular growth, proliferation, development, cell death, and survival [7]. All these processes are physiologically significant for determining epithelial cell fate, ensuring epithelial cell functions, and fine-tuning epithelial immune responses against infection [85].

## Conclusions

Combined with current literature, our data show that many functions of several miRNAs seem to be highly conserved across species. However, while many miRNAs can have similar functions, specific miRNAs also can have different functions in PMECs. We suppose the identified miRNAs have critical roles in pathogen recognition and initiation of local and systemic immune responses in PMECs. Altogether, our results strongly suggest that DE miRNAs in PMECs contribute to pathogenesis of porcine mastitis induced by *E. coli.* The detection of several known and novel miRNAs in context with predicted target genes in PMECs is another indicator for the immune competence of these cells and provides a strong impulse to further examine porcine mammary gland immune defense mechanisms.

## Methods

### Cell culture and pathogen challenge

PMECs are originated from our previous study [7]. Therefore, cell cultures were established from mammary glands of three lactating sows of commercial herds. Animal care and tissue collection was performed in compliance with the German Law of Animal Protection. The experimental protocol was approved by the Animal Care Committee of the Leibniz Institute for Farm Animal Biology, Dummerstorf, Germany. Animals were exsanguinated following electronarcosis and dissected. Cryopreserved mammary epithelial cells were thawed and resuspended in complete medium consisting of Dulbecco’s Modified Eagle Medium/Nutrient Mixture F-12 (DMEM/F12, PAN Biotech, Aidenbach Germany), 10% fetal bovine serum (FBS, PAA, Cölbe, Germany), 1% Antibiotic/Antimycotic Solution (APS, 10,000 U/ml penicillin, 10 mg/ml streptomycin sulphate, 25 μg/ml amphotericin B, PAA), 10 μg/ml insulin (Sigma-Aldrich, Taufkirchen, Germany) and 1 μg/ml hydrocortisone (Sigma-Aldrich).

*E. coli* strain (gMEc240, sequence type 101, phylogroup B1, C+) used for this study is an isolate from milk of PDS-positive sows and was provided by the Institute of Microbiology and Epizootics, Department of Veterinary at the Freie Universität Berlin, Berlin, Germany. *E. coli* was grown in brain-heart-infusion-broth (BHB, Oxoid, Wesel, Germany) at 37 °C to the logarithmic phase of culture growth (Optical Density at 600 nm [OD_600_] 0.5 ~ 5 × 10^7^/ml). Bacteria were heat-inactivated at 80 °C for 1 h, spun down at 3000 rpm for 15 min, washed twice with Dulbecco’s Modified Eagle Medium/Nutrient Mixture F-12 (DMEM/F12) medium and resuspended herein at a density of 1 × 10^8^/ml.

The experimental design was identical with the procedure as described before [7]. Briefly, approximately 4.4 × 10^5^ cells from each 3 individual (3 biological replicates) were seeded and cultured in collagen-coated 6-well plates (1:10 collagen R in destilled water, Menal, Emmendingen, Germany) in complete medium without APS (three technical replicates per individual and treatment condition). After 24 h, medium was changed. Within the next 48 h after seeding, cells reached about 90% confluency. Then PMECs were challenged with 10^7^/ml heat-inactivated *E. coli* for 3 h and for 24 h. Unchallenged cells were used as a negative control. After incubation periods, medium was discarded, and cells were washed three times with phosphate buffered saline (PBS, PAA) to remove the bacteria. Afterwards, cells were collected for isolation of total RNA and small RNA.

### RNA isolation

Total RNA was isolated from each of the 27 samples using the TRI® reagent (Sigma-Aldrich) and extracted with phenol-chloroform according to the manufacturer’s instructions. Isolated RNA was purified using RNeasy Mini Kit (Qiagen, Hilden, Germany), and contaminating DNA was removed by DNase I digestion (Qiagen). Isolation and enrichment of small RNA was performed by using a miRNeasy kit and an RNeasy MinElute Cleanup kit (both from Qiagen) according to the manufacturer’s protocols. RNA integrity and quantity were assessed with an Agilent 2100 Bioanalyzer using an RNA 6000 Nano kit for total RNA and a separate kit for small RNA (both from Agilent Technologies Inc., Santa Clara, CA, USA). The mean RIN and standard deviation for the samples used in the analysis was 9.72 ± 0.24.

### microRNA microarray analysis and statistics

microRNA expression profiling was performed using hybridization to GeneChip miRNA 3.0 Arrays (Affymetrix Inc., Santa Clara, CA, USA) according to the manufacturer’s recommendations. The array has a very high sensitivity and specificity and offers 100% coverage of miRBase 17 (www.mirbase.org) by a one-color approach. In addition, the array contains 16,772 entries representing hairpin precursor, total probe set 19,724 for detection of 1733 mature miRNA, 2216 human snoRNA and scaRNA products in 153 species, and provides a greater than 3-log dynamic range with higher than 95% reproducibility and 85% transcript detection at 1.0 amol for a total RNA input of 130–500 ng.

In total 27 small RNA samples were isolated. Equivalent amount of small RNA from three technical replicates per individual was pooled. In total 9 pooled samples of small RNA were used for further experiment. An amount of 200 ng of each small RNA sample pool was labeled with the FlashTag Biotin HSR RNA Labeling kit (Genisphere, Hatfield, PA). Labeled RNA was hybridized for 16 h to the miRNA arrays according to the manufacturer’s recommendations. A total of nine microarrays were obtained. Afterwards, arrays were washed and stained in a Fluidics Station 450 (Affymetrix), and scanned on the G3000 GeneArray Scanner (Affymetrix). Pre-processing of raw expression data was done using the Expression Console™ software (Affymetrix). Robust multi-array average (RMA) background correction, log-2 transformation, quantile normalization and statistical analysis were performed using SAS 9.4 containing JMP Genomics 7 (SAS Institute, Cary, NC, USA). The ANOVA procedure in JMP genomics 7 was performed to determine relative changes in miRNA levels, including effects mediated by 3 experimental group (before (0 h) and after challenged (3 h and 24 h) with each 3 replicates of each experimental group. Alterations in transcript abundances were considered to be statistically significant at *p* < 0.05. Subsequently, data were filtered by fold change >1.5 or < −1.5. The miRNA microarray data were deposited in the Gene Expression Omnibus (GEO) public repository (GEO accession number GSE88870: GSM2350436 -GSM2350444).

### Prediction and functional annotation of miRNA target genes

We used our previous microarray-based mRNA expression data to integrate with the DE miRNAs and scan for potential target genes [7]. TargetScan was used to detect predicted target genes based on seed complementarity on both 3′-, 5′-UTR and coding sequences of the porcine mRNA sequences (*Sus scrofa* 10.2 download from NCBI: http://www.ncbi.nlm.nih.gov/ on 1.9.2015) and miRNA sequences [86]. RNAhybrid software (http://bibiserv.techfak.uni-bielefeld.de/rnahybrid) was used for direct prediction of multiple, energetically most favorable potential binding sites of our differentially expressed miRNAs [87, 88]. microRNAs were tested due to the following default parameters: (i) number of hits per target, 1; (ii) energy cutoff, −25 kcal/mol; (iii) maximal internal or bulge loop size per side, 4. Pearson correlation of miRNA and mRNA expression levels was calculated based on the predicted miRNA-mRNA relationships. We selected only negative correlation pairs of miRNA-mRNA with *p* < 0.05. The most affected biological processes and molecular functions as well as enriched KEGG pathways of predicted target genes were determined using the Database for Annotation, Visualization and Integrated Discovery 6.7 (DAVID) [89]. In addition, the top canonical pathways, miRNA targets are involved in, were identified using the Ingenuity Pathway Analysis software (*p* ≤ 0.05, Fisher’s exact test; IPA, Ingenuity Systems, Redwood City, CA, USA).

### Validation of microRNA microarray results by Fluidigm real-time quantitative PCR

Transcript levels of seven miRNAs were measured by RT-qPCR using the Fluidigm BioMark HD System (Fluidigm Corporation, San Francisco, CA, USA). First, 200 ng of miRNA was converted to cDNA using the Megaplex RT Primers, Human Pool Kit v2.1/v3.0 (Thermo Fisher Scientific, Waltham, MA, USA). The pre-amplification sample mixtures were prepared containing 1.25 μl of diluted cDNA, 2.5 μl of 2× Taqman pre-amplification master mix (Applied Biosystems, Waltham, MA, USA), and 0.5 μl of the primer pool (200 nmol each primer/μl; Promega, Mannheim, Germany; Additional file 6). The pre-amplification PCR program consisted of a 10 min 95 °C denaturation step and 14 cycles of 15 s at 95 °C and 4 min at 60 °C. The pre-amplified reactions were diluted 10 times in Tris-EDTA (TE) buffer. Five microliters from sample mix containing pre-amplified cDNA, pooled primers and EvaGreen master mix (Fluidigm BioMark) were combined as outlined in the manufacturer’s protocol. The 48.48 dynamic array chip (Fluidigm Corporation) was first primed with control line fluid and then loaded with the sample and assay mixtures via the appropriate inlets using an IFC controller. The array chip was placed in the Fluidigm BioMark Instrument for PCR at 95 °C for 10 min, followed by 30 cycles at 95 °C for 15 s and 60 °C for 1 min. The samples were investigated in duplicate, and data were analyzed with RT-PCR analysis software in the BioMark HD instrument. The mean Ct values of the different miRNAs were normalized to U6 snRNA and miR-24-3p (internal controls), and then subjected to analysis using the 2^−ΔΔCt^ method [90]. Both internal controls were selected, because their transcript levels were most stable over time and treatment in PMECs. In addition, U6 snRNA is known as one of the most commonly used internal control for the normalization of miRNA qPCR data.

## Additional files

Additional file 1: Significantly DE miRNA probesets from microarray analysis in PMECs at 3 hpc and 24 hpc with *E. coli* compared with unchallenged controls. (XLS 78 kb)

Additional file 2: Significantly DE miRNA probesets and their predicted target genes (negatively correlated) from microarray analysis in PMECs at 3 hpc with *E. coli* compared with unchallenged controls. (XLS 82 kb)

Additional file 3: Significantly DE miRNAs and their predicted target genes (negatively correlated) from microarray analysis in PMECs at 24 hpc with *E. coli* compared with unchallenged controls. (XLS 208 kb)

Additional file 4: Significantly enriched GO functional annotation of predicted target genes in PMECs at 3 hpc and 24 hpc with *E. coli*. (XLS 92 kb)

Additional file 5: KEGG pathways and canonical pathways significantly enriched by predicted target genes in PMECs at 3 hpc and 24 hpc with *E. coli*. (XLS 34 kb)

Additional file 6: Verification of selected miRNA expression by RT-qPCR. (XLS 29 kb)

## Abbreviations

DE: Differentially expressed
miRNA: microRNA
PDS: Postpartum dysgalactia syndrome
PMECs: Porcine mammary epithelial cells

## References

[1] Gerjets I, Kemper N (2009). Coliform mastitis in sows: a review. J Swine Health Prod.

[2] López J, Ubiergo A. Often overlooked: subclinical mastitis, in Pig Progress. Reed Business Information, Doetinchem, Netherlands 2005, 21:12–14.

[3] Contreras GA, Rodríguez JM (2011). Mastitis: comparative etiology and epidemiology. J Mammary Gland Biol Neoplasia.

[4] Gerjets I, Traulsen I, Reiners K, Kemper N (2011). Comparison of virulence gene profiles of Escherichia coli isolates from sows with coliform mastitis and healthy sows. Vet Microbiol.

[5] Kemper N, Bardehle D, Lehmann J, Gerjets I, Looft H, Preissler R (2013). The role of bacterial pathogens in coliform mastitis in sows. Berl Munch Tierarztl Wochenschr.

[6] Elmore RG, Martin CE, Berg P (1978). Absorption of Escherichia coli endotoxin from the mammmary glands and uteri of early postpartum sows and gilts. Theriogenol.

[7] Jaeger A, Bardehle D, Oster M, Günther J, Muráni E, Ponsuksili S, Wimmers K, Kemper N (2015). Gene expression profiling of porcine mammary epithelial cells after challenge with Escherichia coli and Staphylococcus aureus in vitro. Vet Res.

[8] Lu LF, Liston A (2009). MicroRNA in the immune system, microRNA as an immune system. Immunology.

[9] Ambros V, Chen X (2007). The regulation of genes and genomes by small RNAs. Development.

[10] Zhou R, O'Hara SP, Chen XM (2011). MicroRNA regulation of innate immune responses in epithelial cells. Cell Mol Immunol.

[11] Imler JL, Hoffmann JA (2001). Toll receptors in innate immunity. Trends Cell Biol.

[12] Jin W, Ibeagha-Awemu E, Liang G, Beaudoin F, Zhao X, Guan LL (2014). Transcriptome microRNA profiling of bovine mammary epithelial cells challenged with Escherichia coli or Staphylococcus aureusbacteria reveals pathogen directed microRNA expression profiles. BMC Genomics.

[13] Sun J, Aswath K, Schroeder SG, Lippolis JD, Reinhardt TA, Sonstegard TS (2015). MicroRNA expression profiles of bovine milk exosomes in response to Staphylococcus aureus infection. BMC Genomics.

[14] Wang XG, Huang JM, Feng MY, Ju ZH, Wang CF, Yang GW, Yuan JD, Zhong JF (2014). Regulatory mutations in the A2M gene are involved in the mastitis susceptibility in dairy cows. Anim Genet.

[15] Chen L, Liu X, Li Z, Wang H, Liu Y, He H, Yang J, Niu F, Wang L, Guo J (2014). Expression differences of miRNAs and genes on NF-κB pathway between the healthy and the mastitis Chinese Holstein cows. Gene.

[16] Gigli I, Maizon DO (2013). microRNAs and the mammary gland: a new understanding of gene expression. Genet Mol Biol.

[17] Günther J, Esch K, Poschadel N, Petzl W, Zerbe H, Mitterhuemer S, Blum H, Seyfert HM (2011). Comparative kinetics of Escherichia coli- and Staphylococcus aureus-specific activation of key immune pathways in mammary epithelial cells demonstrates that S. aureus elicits a delayed response dominated by interleukin-6 (IL-6) but not by IL-1A or tumor necrosis factor alpha. Infect Immun.

[18] Lawless N, Foroushani AB, McCabe MS, O’Farrelly C, Lynn DJ (2013). Next generation sequencing reveals the expression of a unique miRNA profile in response to a gram-positive bacterial infection. PLoS One.

[19] Pal B, Chen Y, Bert A, Hu Y, Sheridan JM, Beck T, Shi W, Satterley K, Jamieson P, Goodall GJ (2015). Integration of microRNA signatures of distinct mammary epithelial cell types with their gene expression and epigenetic portraits. Breast Cancer Res.

[20] Zhu QY, Liu Q, Chen JX, Lan K, Ge BX (2010). MicroRNA-101 targets MAPK phosphatase-1 to regulate the activation of MAPKs in macrophages. J Immunol.

[21] Fordham JB, Naqvi AR, Nares S (2015). Regulation of miR-24, miR-30b, and miR-142-3p during macrophage and dendritic cell differentiation potentiates innate immunity. J Leukoc Biol.

[22] Qi J, Qiao Y, Wang P, Li S, Zhao W, Gao C (2012). microRNA-210 negatively regulates LPS-induced production of proinflammatory cytokines by targeting NF-κB1 in murine macrophages. FEBS Lett.

[23] Fan G, Jiang X, Wu X, Fordjour PA, Miao L, Zhang H, Zhu Y, Gao X (2016). Anti-inflammatory activity of tanshinone IIA in LPS-stimulated RAW264.7 macrophages via miRNAs and TLR4-NF-κB pathway. Inflammation.

[24] Jiang X, Li N (2011). Induction of MiR-17-3p and MiR-106a [corrected] by TNFα and LPS. Cell Biochem Funct.

[25] Staedel C, Darfeuille F (2013). MicroRNAs and bacterial infection. Cell Microbiol.

[26] Zhang T, Yu J, Zhang Y, Li L, Chen Y, Li D, Liu F, Zhang CY, Gu H, Zen K (2014). Salmonella enterica serovar enteritidis modulates intestinal epithelial miR-128 levels to decrease macrophage recruitment via macrophage colony-stimulating factor. J Infect Dis.

[27] Herbert C, Sebesfi M, Zeng QX, Oliver BG, Foster PS, Kumar RK (2015). Using multiple online databases to help identify microRNAs regulating the airway epithelial cell response to a virus-like stimulus. Respirology.

[28] Schmitt MJ, Margue C, Behrmann I, Kreis S (2013). MiRNA-29: a microRNA family with tumor-suppressing and immune-modulating properties. Curr Mol Med.

[29] Zhang Q, Guo XK, Gao L, Huang C, Li N, Jia X, Liu W, Feng WH (2014). MicroRNA-23 inhibits PRRSV replication by directly targeting PRRSV RNA and possibly by upregulating type I interferons. Virology.

[30] Etebari K, Asgari S (2013). Conserved microRNA miR-8 blocks activation of the Toll pathway by upregulating Serpin 27 transcripts. RNA Biol.

[31] Wu W, He C, Liu C, Cao AT, Xue X, Evans-Marin HL, Sun M, Fang L, Yao S, Pinchuk IV (2015). miR-10a inhibits dendritic cell activation and Th1/Th17 cell immune responses in IBD. Gut.

[32] Lee J, Park EJ, Yuki Y, Ahmad S, Mizuguchi K, Ishii KJ, Shimaoka M, Kiyono H (2015). Profiles of microRNA networks in intestinal epithelial cells in a mouse model of colitis. Sci Rep.

[33] Salilew-Wondim D, Ibrahim S, Gebremedhn S, Tesfaye D, Heppelmann M, Bollwein H, Pfarrer C, Tholen E, Neuhoff C, Schellander K (2016). Clinical and subclinical endometritis induced alterations in bovine endometrial transcriptome and miRNome profile. Genomics.

[34] Ye FG, Song CG, Cao ZG, Xia C, Chen DN, Chen L, Li S, Qiao F, Ling H, Yao L (2015). Cytidine Deaminase axis modulated by miR-484 differentially regulates cell proliferation and chemoresistance in breast cancer. Cancer Res.

[35] Krishnan P, Ghosh S, Wang B, Li D, Narasimhan A, Berendt R, Graham K, Mackey JR, Kovalchuk O, Damaraju S (2015). Next generation sequencing profiling identifies miR-574-3p and miR-660-5p as potential novel prognostic markers for breast cancer. BMC Genomics.

[36] Achari C, Winslow S, Ceder Y, Larsson C (2014). Expression of miR-34c induces G2/M cell cycle arrest in breast cancer cells. BMC Cancer.

[37] Wu ZS, Wu Q, Wang CQ, Wang XN, Wang Y, Zhao JJ, Mao SS, Zhang GH, Zhang N, Xu XC (2010). MiR-339-5p inhibits breast cancer cell migration and invasion in vitro and may be a potential biomarker for breast cancer prognosis. BMC Cancer.

[38] Tsai KW, Leung CM, Lo YH, Chen TW, Chan WC, Yu SY, Tu YT, Lam HC, Li SC, Ger LP (2016). Arm selection preference of MicroRNA-193a varies in breast cancer. Sci Rep.

[39] Ni F, Gui Z, Guo Q, Hu Z, Wang X, Chen D, Wang S (2016). Downregulation of miR-362-5p inhibits proliferation, migration and invasion of human breast cancer MCF7 cells. Oncol Lett.

[40] Yang W, Zerbe H, Petzl W, Brunner RM, Günther J, Draing C, von Aulock S, Schuberth HJ, Seyfert HM (2008). Bovine TLR2 and TLR4 properly transduce signals from Staphylococcus aureus and E. coli, but S. aureus fails to both activate NF-kappaB in mammary epithelial cells and to quickly induce TNFalpha and interleukin-8 (CXCL8) expression in the udder. Mol Immunol.

[41] Zhang D, Cao X, Li J, Zhao G (2015). MiR-210 inhibits NF-κB signaling pathway by targeting DR6 in osteoarthritis. Sci Rep.

[42] Peng P, Li Z, Liu X (2015). Reduced expression of miR-23a suppresses A20 in TLR-stimulated macrophages. Inflammation.

[43] Naqvi AR, Fordham JB, Nares S (2015). miR-24, miR-30b, and miR-142-3p regulate phagocytosis in myeloid inflammatory cells. J Immunol.

[44] Hicks JA, Yoo D, Liu HC (2013). Characterization of the microRNAome in porcine reproductive and respiratory syndrome virus infected macrophages. PLoS One.

[45] Yin H, Sun Y, Wang X, Park J, Zhang Y, Li M, Yin J, Liu Q, Wei M (2015). Progress on the relationship between miR-125 family and tumorigenesis. Exp Cell Res.

[46] Liu L, Li Q, Xiao X, Wu C, Gao R, Peng C, Li D, Zhang W, Du T, Wang Y (2016). miR-1934, downregulated in obesity, protects against low-grade inflammation in adipocytes. Mol Cell Endocrinol.

[47] Meng F, Zhang Z, Chen W, Huang G, He A, Hou C, Long Y, Yang Z, Zhang Z, Liao W (2016). MicroRNA-320 regulates matrix metalloproteinase-13 expression in chondrogenesis and interleukin-1β-induced chondrocyte responses. Osteoarthr Cartil.

[48] Kong Y, Wu J, Yuan L (2014). MicroRNA expression analysis of adult-onset Drosophila Alzheimer’s disease model. Curr Alzheimer Res.

[49] Sibbesen NA, Kopp KL, Litvinov IV, Jønson L, Willerslev-Olsen A, Fredholm S, Petersen DL, Nastasi C, Krejsgaard T, Lindahl LM (2015). Jak3, STAT3, and STAT5 inhibit expression of miR-22, a novel tumor suppressor microRNA, in cutaneous T-Cell lymphoma. Oncotarget.

[50] Ma S, Liu M, Xu Z, Li Y, Guo H, Ge Y, Liu Y, Zheng D, Shi J (2016). A double feedback loop mediated by microRNA-23a/27a/24-2 regulates M1 versus M2 macrophage polarization and thus regulates cancer progression. Oncotarget.

[51] Dai N, Zhong ZY, Cun YP, Qing Y, Chen C, Jiang P, Li MX, Wang D (2013). Alteration of the microRNA expression profile in human osteosarcoma cells transfected with APE1 siRNA. Neoplasma.

[52] Gougelet A, Pissaloux D, Besse A, Perez J, Duc A, Dutour A, Blay JY, Alberti L (2011). Micro-RNA profiles in osteosarcoma as a predictive tool for ifosfamide response. Int J Cancer.

[53] Chen S, Sun KX, Liu BL, Zong ZH, Zhao Y (2016). MicroRNA-505 functions as a tumor suppressor in endometrial cancer by targeting TGF-α. Mol Cancer.

[54] Song H, Zhang Y, Liu N, Wan C, Zhang D, Zhao S, Kong Y, Yuan L (2016). miR-92b regulates glioma cells proliferation, migration, invasion, and apoptosis via PTEN/Akt signaling pathway. J Physiol Biochem.

[55] zur Bruegge J, Backes C, Gölz G, Hemmrich-Stanisak G, Scharek-Tedin L, Franke A, Alter T, Einspanier R, Keller A, Sharbati S (2016). MicroRNA response of primary human macrophages to arcobacter butzleri infection. Eur J Microbiol Immunol (Bp).

[56] Liston A, Papadopoulou AS, Danso-Abeam D, Dooley J (2012). MicroRNA-29 in the adaptive immune system: setting the threshold. Cell Mol Life Sci.

[57] Salama A, Fichou N, Allard M, Dubreil L, De Beaurepaire L, Viel A, Jégou D, Bösch S, Bach JM (2014). MicroRNA-29b modulates innate and antigen-specific immune responses in mouse models of autoimmunity. PLoS One.

[58] Chen W, Ou HS (2016). Regulation of miR-24 on vascular endothelial cell function and its role in the development of cardiovascular disease. Sheng Li Xue Bao.

[59] Warg LA, Oakes JL, Burton R, Neidermyer AJ, Rutledge HR, Groshong S, Schwartz DA, Yang IV (2012). The Role of the E2F1 transcription factor in the innate immune response to systemic LPS. Am J Physiol Lung Cell Mol Physiol.

[60] Trakooljul N, Hicks JA, Liu HC (2010). Identification of target genes and pathways associated with chicken microRNA miR-143. Anim Genet.

[61] Pierdomenico M, Cesi V, Cucchiara S, Vitali R, Prete E, Costanzo M, iM A, Oliva S, Stronati L (2016). NOD2 is regulated By Mir-320 in physiological conditions but this control is altered in inflamed tissues of patients with inflammatory bowel disease. Inflamm Bowel Dis.

[62] Suárez Y, Wang C, Manes TD, Pober JS (2010). Cutting edge: TNF-induced microRNAs regulate TNF-induced expression of E-selectin and intercellular adhesion molecule-1 on human endothelial cells: feedback control of inflammation. J Immunol.

[63] Hui AB, Lin A, Xu W, Waldron L, Perez-Ordonez B, Weinreb I, Shi W, Bruce J, Huang SH, O'Sullivan B (2013). Potentially prognostic miRNAs in HPV-associated oropharyngeal carcinoma. Clin Cancer Res.

[64] Lee GJ, Hyun S (2014). Multiple targets of the microRNA miR-8 contribute to immune homeostasis in Drosophila. Dev Comp Immunol.

[65] Bolin K, Rachmaninoff N, Moncada K, Pula K, Kennell J, Buttitta L (2016). miR-8 modulates cytoskeletal regulators to influence cell survival and epithelial organization in Drosophila wings. Dev Biol.

[66] Guan J, Wang G, Tam LS, Kwan BC, Li EK, Chow KM, Li PK, Szeto CC (2012). Urinary sediment ICAM-1 level in lupus nephritis. Lupus.

[67] Conti A, Romeo SG, Cama A, La Torre D, Barresi V, Pezzino G, Tomasello C, Cardali S, Angileri FF, Polito F (2016). MiRNA expression profiling in human gliomas: upregulated miR-363 increases cell survival and proliferation. Tumour Biol.

[68] Hatano K, Kumar B, Zhang Y, Coulter JB, Hedayati M, Mears B, Ni X, Kudrolli TA, Chowdhury WH, Rodriguez R (2015). A functional screen identifies miRNAs that inhibit DNA repair and sensitize prostate cancer cells to ionizing radiation. Nucleic Acids Res.

[69] Huang Y, Chuang AY, Ratovitski EA (2011). Phospho-ΔNp63α/miR-885-3p axis in tumor cell life and cell death upon cisplatin exposure. Cell Cycle.

[70] Liang Y, Duan L, Xiong J, Zhu W, Liu Q, Wang D, Liu W, Li Z, Wang D (2016). E2 regulates MMP-13 via targeting miR-140 in IL-1β-induced extracellular matrix degradation in human chondrocytes. Arthritis Res Ther.

[71] Zhang T, Zou P, Wang T, Xiang J, Cheng J, Chen D, Zhou J (2016). Down-regulation of miR-320 associated with cancer progression and cell apoptosis via targeting Mcl-1 in cervical cancer. Tumour Biol.

[72] Ma G, Zhang F, Dong X, Wang X, Ren Y (2016). Low expression of microRNA-202 is associated with the metastasis of esophageal squamous cell carcinoma. Exp Ther Med.

[73] Li YJ, Dong M, Kong FM, Zhou JP, Liang D, Xue HZ (2016). MicroRNA-371-5p targets SOX2 in gastric cancer. Oncotarget.

[74] Zhang H, Zhu X, Li N, Li D, Sha Z, Zheng X, Wang H (2015). miR-125a-3p targets MTA1 to suppress NSCLC cell proliferation, migration, and invasion. Acta Biochim Biophys Sin Shanghai.

[75] Liu X, Wang S, Yuan A, Yuan X, Liu B (2016). MicroRNA-140 represses glioma growth and metastasis by directly targeting ADAM9. Oncol Rep.

[76] Huang H, Jiang Y, Wang Y, Chen T, Yang L, He H, Lin Z, Liu T, Yang T, Kamp DW (2015). miR-5100 promotes tumor growth in lung cancer by targeting Rab6. Cancer Lett.

[77] Shang C, Hong Y, Guo Y, Liu YH, Xue YX (2016). miR-128 regulates the apoptosis and proliferation of glioma cells by targeting RhoE. Oncol Lett.

[78] Zheng WW, Zhou J, Zhang CH, Liu XS (2016). MicroRNA-216b is downregulated in hepatocellular carcinoma and inhibits HepG2 cell growth by targeting Forkhead box protein M1. Eur Rev Med Pharmacol Sci.

[79] Perdomo C, Campbell JD, Gerrein J, Tellez CS, Garrison CB, Walser TC, Drizik E, Si H, Gower AC, Vick J (2013). MicroRNA 4423 is a primate-specific regulator of airway epithelial cell differentiation and lung carcinogenesis. Proc Natl Acad Sci U S A.

[80] Li Y, Tang X, He Q, Yang X, Ren X, Wen X, Zhang J, Wang Y, Liu N, Ma J (2016). Overexpression of mitochondria mediator gene TRIAP1 by miR-320b loss is associated with progression in nasopharyngeal carcinoma. PLoS Genet.

[81] Huat TJ, Khan AA, Abdullah JM, Idris FM, Jaafar H (2015). MicroRNA expression profile of neural progenitor-like cells derived from rat bone marrow mesenchymal stem cells under the influence of IGF-1, bFGF and EGF. Int J Mol Sci.

[82] Floyd DH, Zhang Y, Dey BK, Kefas B, Breit H, Marks K, Dutta A, Herold-Mende C, Synowitz M, Glass R (2014). Novel anti-apoptotic microRNAs 582-5p and 363 promote human glioblastoma stem cell survival via direct inhibition of caspase 3, caspase 9, and Bim. PLoS One.

[83] Chen K, Shi W (2016). Autophagy regulates resistance of non-small cell lung cancer cells to paclitaxel. Tumour Biol.

[84] Stiuso P, Potenza N, Lombardi A, Ferrandino I, Monaco A, Zappavigna S, Vanacore D, Mosca N, Castiello F, Porto S (2015). MicroRNA-423-5p promotes autophagy in cancer cells and is increased in serum from hepatocarcinoma patients treated with sorafenib. Mol Ther Nucleic Acids.

[85] Liu J, Drescher KM, Chen XM (2009). MicroRNAs and epithelial immunity. Int Rev Immunol.

[86] Lewis BP, Burge CB, Bartel DP (2005). Conserved seed pairing, often flanked by adenosines, indicates that thousands of human genes are microRNA targets. Cell.

[87] Krüger J, Rehmsmeier M (2006). RNAhybrid: microRNA target prediction easy, fast and flexible. Nucleic Acids Res.

[88] Rehmsmeier M, Steffen P, Hochsmann M, Giegerich R (2004). Fast and effective prediction of microRNA/target duplexes. RNA.

[89] Huang da W, Sherman BT, Lempicki RA (2009). Systematic and integrative analysis of large gene lists using DAVID bioinformatics resources. Nat Protoc.

[90] Livak KJ, Schmittgen TD (2001). Analysis of relative gene expression data using real-time quantitative PCR and the 2(−Delta Delta C(T)) Method. Methods.

